# Electrospun Blank Nanocoating for Improved Sustained Release Profiles from Medicated Gliadin Nanofibers

**DOI:** 10.3390/nano8040184

**Published:** 2018-03-22

**Authors:** Xinkuan Liu, Wenyi Shao, Mingyi Luo, Jiayin Bian, Deng-Guang Yu

**Affiliations:** School of Material Science and Engineering, University of Shanghai for Science and Technology, Shanghai 200093, China; swyswy111@163.com (W.S.); minmin_98930@163.com (M.L.); bianjyusst@163.com (J.B.)

**Keywords:** medicated nanofiber, nanocoating, coaxial electrospinning, structural nanocomposite, sustained release, poorly water-soluble drug

## Abstract

Nanomaterials providing sustained release profiles are highly desired for efficacious drug delivery. Advanced nanotechnologies are useful tools for creating elaborate nanostructure-based nanomaterials to achieve the designed functional performances. In this research, a modified coaxial electrospinning was explored to fabricate a novel core-sheath nanostructure (nanofibers F2), in which a sheath drug-free gliadin layer was successfully coated on the core ketoprofen (KET)-gliadin nanocomposite. A monolithic nanocomposite (nanofibers F1) that was generated through traditional blending electrospinning of core fluid was utilized as a control. Scanning electron microscopy demonstrated that both nanofibers F1 and F2 were linear. Transmission electron microscopy verified that nanofibers F2 featured a clear core-sheath nanostructure with a thin sheath layer about 25 nm, whereas their cores and nanofibers F1 were homogeneous KET-gliadin nanocomposites. X-ray diffraction patterns verified that, as a result of fine compatibility, KET was dispersed in gliadin in an amorphous state. In vitro dissolution tests demonstrated that the thin blank nanocoating in nanofibers F2 significantly modified drug release kinetics from a traditional exponential equation of nanofibers F1 to a zero-order controlled release model, linearly freeing 95.7 ± 4.7% of the loaded cargoes over a time period of 16 h.

## 1. Introduction

Oral delivery is the most popular drug administration route for patients because of its convenience. However, therapeutic effects of drugs constantly present a large gap that requires improvement. In the half past century, numerous efforts have been spent on the development of medicated materials that can provide drug sustained drug release profiles, which in turn maintain a long period of drug blood concentration for “effective, safe and convenient” drug delivery goals [1,2]. Thus, oral dosage forms that can provide a zero-order drug sustained release profile (the best sustained release model) are highly desired. 

With the surge of nanoscience from the 1990s, medicated nanomaterials have drawn increasing attention for furnishing all kinds of drug controlled release profiles [3,4]. As for drug sustained release nanomaterials, physical and chemical properties of exploited drug carriers often play dependent roles. The traditional strategy is that drugs are homogeneously distributed over matrices to form medicated nanoproducts. Insoluble or biodegradable properties of matrices can cause the sustained release of encapsulated drug molecules through diffusion or erosion [5,6]. However, these monolithic medicated nanomaterials present two essential weaknesses. One weakness is the uncontrollable initial burst releases resulted from the huge surface area of nanomaterials, which can cause unsafe blood drug concentration higher than the maximum safety standard. The other weakness is long periods of late tailing-off release, which often results in a blood drug concentration that is lower than the minimum effective concentration [7,8]. Thus, at present, more studies focus on elaborate complex nanostructures that can effectively eliminate the initial burst effect and late tailing-off phenomena [9,10,11]. 

Core-sheath nanostructures, with one section (core) being completely encapsulated by another section (sheath), have acted as one of the most powerful platforms to support the development of functional nanomaterials during the past decades [12,13]. Many novel strategies stemming from this structure have been demonstrated to be useful for designing and developing new drug sustained release nanoparticles, liposomes, solid-lipid nanospheres, and nanofibers. 

Core-sheath nanostructures can be created through top-down techniques, bottom-up techniques, and their combination [12]. Among the different types of nanotechnologies, electrospinning has demonstrated its usefulness in creating nanofibers in a “top-down” and one-step straightforward manner [14,15,16,17,18]. However, the most interesting capability of electrospinning is that it can duplicate nanostructures from a macro-template [19,20,21], i.e., the spinneret’s structure, in a single step and straightforwardly manner. For example, a concentric spinneret and a side-by-side spinneret can be exploited to generate core-sheath and Janus nanofibers [22,23,24,25,26], respectively; a tri-layer coaxial spinneret can be explored to create tri-layer core-sheath nanofibers [27,28]. The facile and easy implementation of electrospinning implies its notable potential for creating nanoproducts with these complex nanostructures in an industrial scale.

Based on the above-mentioned knowledge, in this study, we investigated the possibility of special core-sheath nanostructure, in which a core drug-loaded composite was coated by a thin drug-free blank layer. A modified coaxial process was explored to create these core-sheath nanofibers. Traditionally, the sheath working fluid in coaxial electrospinning must be electrospinnable to ensure a successful coaxial process and the achievement of final structural nanoproducts [29]. However, Yu et al. broke this concept to develop a modified coaxial electrospinning, in which unspinnable solutions (including dilute polymer solutions, solvent, nanosuspensions, and solutions of small molecules) can be explored as sheath working fluid to prepare novel nanostructures [30]. These nanostructures have been demonstrated to be good candidates for pharmaceutical applications [8,25,26,30,31,32,33]. Utilization of unspinnable dilute polymer solutions as sheath fluids can ensure the possibility that ultra-thin sheath layer can be spread continuously and ultra-thinly on the core part of the structural nanofibers. 

To demonstrate the above-mentioned strategy, gliadin and ketoprofen (KET) were selected as drug carrier and active pharmaceutical ingredient, respectively. Gliadin is a plant protein. This compound features fine biocompatibility and biodegradability, as it is derived from natural sources. When compared with numerous pharmaceutical polymeric excipients prepared from chemical synthesis, gliadin poses no concerns about toxic initiator residues or monomer. During the past 10 years, gliadin has been widely investigated for potential applications in food engineering, tissue engineering, and drug delivery. For drug delivery, medicated fibers, microspheres, tablets, and capsules have been reported to be fabricated using spraying, wet spinning, tableting, and electrospinning [33,34,35]. KET is a typical nonsteroidal anti-inflammatory drug and it is frequently exploited for the treatment of rheumatoid arthritis and for its anti-inflammatory effects. KET is an insoluble drug, belonging to Class II drugs of Biopharmaceutical Classification Systems [36]. Sustained release through oral administration of KET is welcomed by patients all over the world [37,38]. 

## 2. Materials and Methods

### 2.1. Materials

KET was bought from the Shanghai Fan-Ke Biology Technology Co., Ltd. (Shanghai, China). Gliadin (extracted from wheat) was bought from Miao-Sheng Biotechnol. Co., Ltd. (Shanghai, China). Solvents 1,1,1,3,3,3-hexafluoro-2-propanol (HFIP, purity 99.0%) and trifluoroacetic acid (TFA) were obtained from Shanghai Zi-Yi Chem. Co., Ltd. (Shanghai, China).

### 2.2. Electrospinning

Sheath blank gliadin solution contained 5% (*w*/*v*) gliadin in HFIP. The core KET-gliadin solution comprised 16% (*w*/*v*) gliadin and 4% (*w*/*v*) KET in a solvent mixture of HFIP and TFA (8:2, *v*:*v*). Methylene blue (5 × 10^−6^ g/mL) was added into the core solution to optimize the experimental parameters.

Core and sheath liquids were placed in separate syringes and were pumped to a home-made concentric spinneret using syringe pumps (KDS100, Cole-Parmer^®^, Vernon Hills, IL, USA). The fibers were deposited on a collector, which was placed vertically under the nozzle of spinneret at a distance of 15 cm. High power supply (ZGF 60 kV/2 mA, Huatian Corp., Wuhan, China) was utilized to furnish a high voltage of 15 kV.

Two kinds of nanofibers were generated. The first type was the monolithic nanofibers F1, which were prepared under a sheath/core fluid flow rate of 0/1 mL/h. The next type was nanofibers F2 with a blank gliadin nanocoating as sheath layer, and which were prepared under a sheath/core fluid flow rate of 0.3/0.7 mL/h.

### 2.3. Morphology and Structure

Nanofiber morphology was evaluated using a Quanta 450 FEG scanning electron microscope (SEM, FEI, Hillsboro, OR, USA) under an applied voltage of 20 kV. The sample was sputter-coated with platinum for 30 s before imaging. The nanofiber’s diameter was estimated using ImageJ software (National Institutes of Health, Bethesda, MD, USA) over 100 places in SEM images. The inner nanofiber structure was determined using an H-800 transmission electron microscope (TEM; Hitachi, Tokyo, Japan).

### 2.4. X-ray Diffraction (XRD) and Attenuated Total Reflectance-Fourier Transform Infrared (ATR-FTIR) Spectroscopy

XRD measurements were conducted on a Bruker X-ray powder diffractometer (Karlsruhe, Germany) with Cu Kα radiation. Analyzed 2θ angles measured 5°–60°. Raw KET and gliadin particles were observed and recorded using an XP-700 polarized microscope (PM, Chang-Fang Optical Co., Ltd., Shanghai, China).

ATR-FTIR spectra were recorded using a Spectrum 100FTIR Spectrometer (PerkinElmer, Billerica, MA, USA). The wavenumber ranged between 4000 cm^−1^ to 700 cm^−1^ at a resolution of 1 cm^−1^ and eight scans were performed for each detection.

### 2.5. In Vitro Dissolution Experiments

KET release from medicated nanofibers was induced by dispersing 20 mg of them into 300 mL of pH 7.0 phosphate buffered saline (PBS) a 37 °C with stirring. At predetermined time points, 5 mL aliquot was drawn and 5 mL fresh PBS was used to compensate the removed aliquot. The samples were filtered through a 0.22 µm filter, after which KET concentrations were determined using a Ultraviolet-Visible (UV) spectrophotometer (Unico Co. Ltd., Shanghai, China).

## 3. Results

### 3.1. Electrospinning

**Figure 1:**
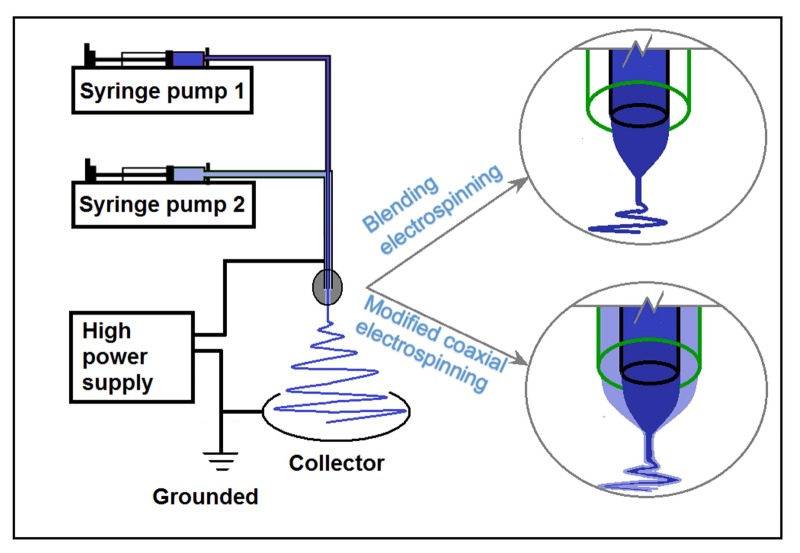
Diagram of blending and modified coaxial electrospinning implemented using the same apparatus.

Figure 1 shows a diagram of blending and modified coaxial electrospinning implemented using the same system. The system consisted of two syringe pumps for driving and metering working fluids, a power supply for providing high voltage, a grounded collector for deposition of solid nanofibers, and a concentric spinneret for guiding the working fluids into the electrical field.

**Figure 2:**
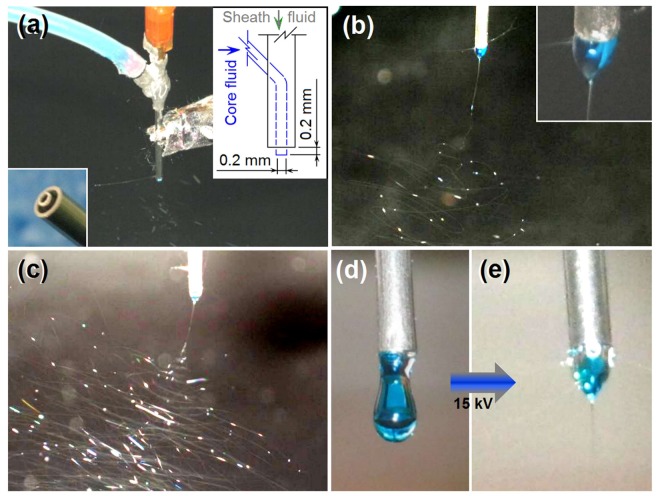
Implementation and observation of electrospinning processes: (**a**) Connection of spinneret with working fluids and power supply; the bottom-left inset shows the spinneret nozzle, whereas the upper-right inset shows a diagram of the flow paths of working fluids; (**b**) Blending electrospinning of ketoprofen (KET)-gliadin core solution; the upper-right inset shows a typical Taylor cone; (**c**) Modified coaxial electrospinning; and, (**d**,**e**) changes from a droplet to a compound Taylor cone.

Figure 2 shows the digital photos that were captured during electrospinning processes. Under the selected experimental conditions, both blending electrospinning of KET-gliadin solution only and modified coaxial electrospinning with an unspinnable dilute gliadin solution surrounding the KET-gliadin fluid ran smoothly and continuously. Figure 2a shows the linkage of spinneret with working fluids and power supply. The core fluid was fed to the spinneret through an elastic silicon tubing, and a syringe containing sheath fluid was directly inserted into the spinneret vertically. High voltage was applied on the working fluids through an alligator clipper. The bottom-left inset shows the spinneret nozzle, in which the inner and outer capillaries presented with a concentric axis, and the inner capillary slightly projected out the outer capillary. The upper-right inset shows a diagram of the flow paths of the sheath fluid (directly from its syringe) and core fluid, which was guided into the spinneret through an obliquely intercalated inner metal capillary.

Figure 2b shows a typical blending electrospinning of KET-gliadin core solution, with the Taylor cone being shown in the upper-right inset. Given the easy infiltration of core solution on metal material, the Taylor cone expanded to the full nozzle instead of only trapped in the inner capillary. Figure 2c shows the working process of the modified coaxial electrospinning, during which a trilogy of Taylor cone, straight fluid jet, and bending and whipping unstable region with enlarged loops was observed. Figure 2d,e show the changes from a core-sheath droplet to a compound Taylor cone under an applied voltage of 15 kV, with a transparent sheath gliadin solution surrounding a methylene blue-marked core solution. 

### 3.2. Nanofibers and Nanocoating

**Figure 3:**
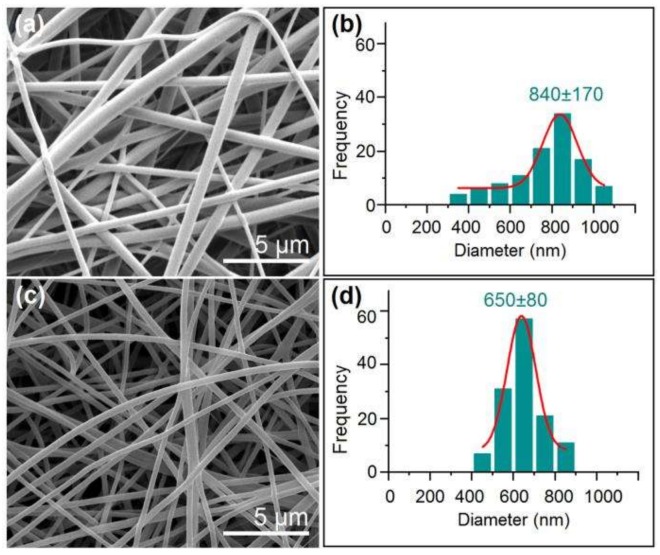
Scanning electron microscope (SEM) images of nanofibers and their size distributions: (**a**) Nanofibers F1 and (**b**) their size distributions; (**c**) Nanofibers F2; and, (**d**) their size distributions.

As anticipated, the core KET-gliadin solution can be individually converted into solid fibers through a single-fluid blending electrospinning. The created nanofibers F1 had exhibited a linear morphology without any discerned beads/spindles-on-a-string phenomena (Figure 3a). These nanofibers showed a broad size distribution of 840 ± 170 nm (Figure 3b). SEM images of nanofibers F2 were linear without any beads/spindles within the nanofiber mats (Figure 3c). However, these nanofibers presented an average diameter of 650 ± 80 nm, showing a better quality than nanofibers F1 in terms of size and size distribution (Figure 3d). 

**Figure 4:**
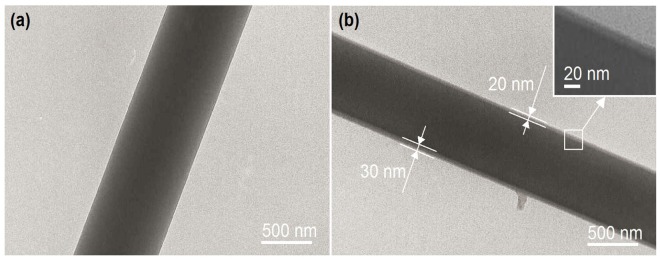
Transmission electron microscope (TEM) images of nanofibers (**a**) F1 and (**b**) F2.

Figure 4 shows the TEM images of fibers. As anticipated, nanofibers F1 showed a uniform gray level (Figure 4a), suggesting that they were homogeneous nanocomposites with drug distributed over the gliadin matrices. In the TEM images of nanofibers F2 (Figure 4b and its upper-right inset), a very thin layer at the sheath sections of nanofibers exhibited a lower gray level than their core sections. The sheath layers featured a thickness between 20 nm to 30 nm. 

### 3.3. Physical State and Compatibility

**Figure 5:**
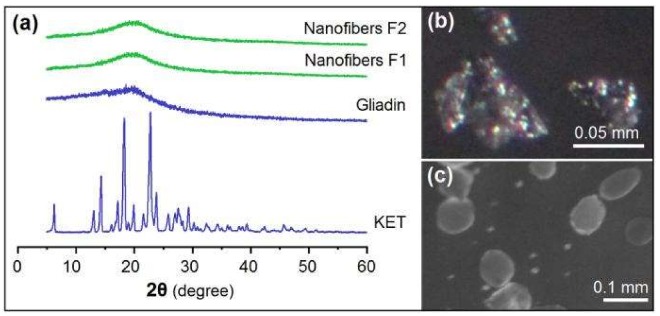
Physical state: (**a**) X-ray Diffraction (XRD) patterns of KET, gliadin, and their nanofibers; (**b**) PM images of KET raw particles and (**c**) gliadin particles.

Figure 5 displays the physical state of raw materials and nanofibers. The sharp reflection peaks in the XRD curve of KET (Figure 5a) and the colorful PM images in Figure 5b imply that raw KET particles are crystalline materials. By contrast, the only halo in the XRD curve of gliadin (Figure 5a) and gray PM images in Figure 5c demonstrate that raw gliadin powders are amorphous materials. Nanofibers F1 and F2 are amorphous composites regardless of their structural characteristics, as suggested by their XRD patterns in Figure 5a. 

**Figure 6:**
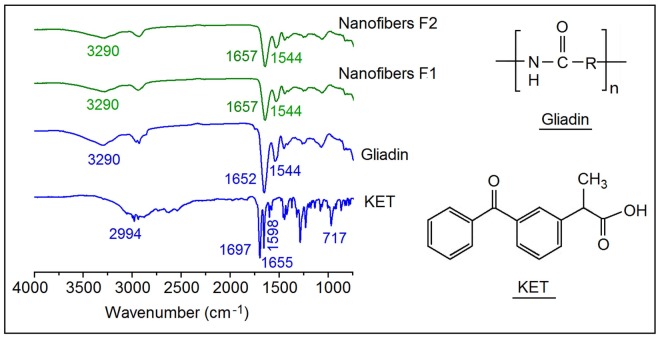
Molecular formulae of gliadin and KET and Attenuated Total Reflectance-Fourier Transform Infrared (ATR-FTIR) spectra of KET, gliadin, and composite nanofibers.

Figure 6 shows the molecular formulae of gliadin and KET. Both of the compounds can act as proton donor and receptor for forming hydrogen bonds. In the ATR-FTIR spectra of KET, two sharp peaks are visible for raw KET particles: one at 1697 cm^−1^, representing the stretching vibration of the carbonyl group in the dimeric carboxylic acid, and the other at 1655 cm^−1^, due to the stretching vibration of the carbonyl group in the ketonic group. The former is a result that KET molecules are bound together in dimmers pure KET powders [28,37]. However, it completely disappeared in the curves of nanofibers F1 and F2. On the other hand, all of the peaks in the fingerprint regions of KET weakened or completely disappeared from the nanofiber spectra, and carbonyl stretching peak of gliadin shifted slightly from 1652 cm^−1^ to 1657 cm^−1^ in the nanocomposites. These phenomena jointly indicate the breakage of KET dimers and the formation of hydrogen bonds between gliadin and KET molecules in their nanofibers. 

### 3.4. Sustained Release Profiles

**Figure 7:**
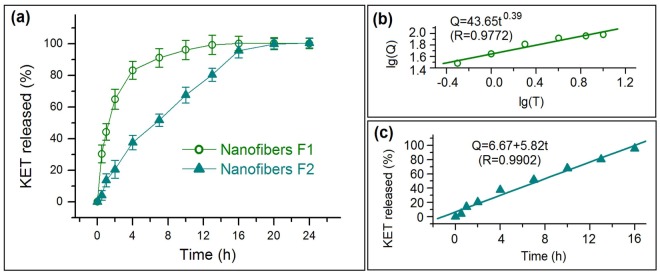
In vitro dissolution tests: (**a**) KET release profiles from nanofibers F1 and F2; (**b**) fitting exponent equation of drug release data from nanofibers F1; (**c**) fitting linear equation of drug release data from nanofibers F2.

Figure 7a illustrates the results of KET in vitro dissolution. Notably, nanofibers F2 can provide better drug sustained release profiles than nanofibers F1 in terms of initial burst release effect, sustained release time period, and tailing-off release phenomenon. Nanofibers F1 nanofibers exhibited a severe initial burst release, with a release content of 30.4 ± 5.7% and 44.3 ± 5.2% for 0.5 and 1 h, respectively. By contrast, 4.1 ± 3.1% and 13.7 ± 4.2% were freed from F2 nanofibers for the same time periods, indicating the complete elimination of initial burst release. After 10 and 16 h, F1 nanofibers released 96.1 ± 5.6% and 100.2 ± 4.5%, respectively. However, nanofibers F2 released 95.7 ± 4.7% and 100.1 ± 3.4% after 16 and 20 h, respectively, indicating that F2 featured a longer sustained release period but a smaller tailing-off release negative phenomenon than F1 nanofibers. 

To fit the drug release data according to the zero-order equation (*Q* = a*t* + b) and Peppas equation [39] (*Q* = k*t^n^*), where *Q* refer to the drug release percentage, *t* denotes release time; k, a, and b are constants, and *n* is the release exponent that indicates the drug release mechanism. For nanofibers F1, their release equation is *Q* = 43.65*t*^0.39^ (*R* = 0.9772) (Figure 7b). The release exponent *n* is 0.39 (which is lower than 0.45), suggesting that the KET released from gliadin matrices was manipulated by a typical Fickian diffusion mechanism. For F2 nanofibers, their release equation is *Q* = 6.67 + 5.82*t* (*R* = 0.9902) (Figure 7c), suggesting a fine zero-order drug-controlled release. 

## 4. Discussion

In this experiment, two approaches were explored to expand the capability of electrospinning in generating complex nanostructures. The first approach involves a homemade spinneret, whereas the other applies unspinnable dilute gliadin solution as sheath working fluid. In electrospinning systems, the spinneret is the most important part [40]. Here, the concentric spinneret can ensure the smooth preparations of both monolithic nanofibers F1 and core-sheath nanofibers F2 through manipulations of the flow rate of working fluids. When the unspinnable sheath solution was switched off, the working process became a traditional single-fluid electrospinning for converting spinnable core solutions into solid nanofibers F1 directly. 

As the numbers of unspinnable liquids are extremely larger than spinnable polymer solutions, exploration of unspinnable liquids will remarkably extend the possibilities in generating nanostructures using one-step electrospinning. In this study, for easy implementation, dilute gliadin solution with a concentration of 5% (*w*/*v*) in HFIP was explored to form a blank nanocoating on the core KET-gliadin medicated nanocomposites. The sheath unspinnable fluid and core spinnable fluid contained the same kind of macromolecules (gliadin) and also the same solvent (HFIP), indicating the high compatibility of the two fluids. In the future, new possibilities can be generated using a similar protocol but with different kinds of polymeric matrices, which are utilized in a combined manner for realizing a final joint functional performance. Diffusion between core and sheath fluids should not be a concern, as this property has been demonstrated to very weak between two parallel working micro-/nanofluids [41]. A similar strategy can also be applied to expand the capability of coaxial electrospraying (a counterpart of coaxial electrospinning) in creating novel nanomaterials in the form of particles [42]. 

Although an unspinnable dilute solution was utilized as sheath working fluid in the modified coaxial process, this choice exerted little influence on the formation of electrospun amorphous medicated nanocomposites. Regardless of the number of fluids that were treated simultaneously during electrospinning, the procedure remains a one-step straightforward and an extremely fast drying process. The uniform distribution state of components in the working solution can be totally propagated into solid nanoproducts. Provided that the components are compatible, a stable composite can be created and maintained. In the present study, KET-gliadin intermolecular hydrogen bonds predominated in both nanofibers F1 and the cores of nanofibers F2. These secondary interactions, together with spatial hindrance effects of gliadin molecules, should effectively prevent the formation of dimers, crystal lattice, and crystal growth of KET.

KET-gliadin interactions should increase the compactness of composites. The sheath-to-core weight ratio (*R*_w_) can be calculated according to solute concentrations (*C*_s_ and *C*_c_ represent solute concentrations in the sheath and core fluids, respectively) in the working fluids and their flow rates (*F*_s_ and *F*_c_ represent the flow rates of sheath and core fluids, respectively), i.e., *R*_w_ = *C*_s_*F*_s_/*C*_c_*F*_c_. In this study, on one hand, *R*_w_ yielded a value of (5% × 0.3)/((16% + 4%) × 0.7) = 0.107. On the other hand, sheath-to-core (*V*_w_) volume ratio can be estimated according to surface areas (S_s_ and S_c_ represent the surface areas of sheath circular ring and core circle, respectively) and to diameters (*r*_f_ and *r*_c_ represent the diameters of the whole nanofibers and core circle section, respectively) measured using TEM owing to the same fiber lengths, i.e., *V*_w_ = *S*_s_/*S*_c_ = (*r*_f_^2^ − *r*_c_^2^)/*r*_c_^2^ = (325^2^ − 300^2^)/300^2^ = 0.174. The sheath layer presented a density that is 61.5% (0.107/0.174 × 100%) of the core section. The favorable secondary interactions between the drug (KET) and carrier (gliadin) molecules through hydrogen bonding should take the charge of a denser core than the blank sheath gliadin nanocoating. Thus, the blank nanocoating exhibited a lower gray level than the core KET-gliadin composite that is in Figure 4b. 

**Figure 8:**
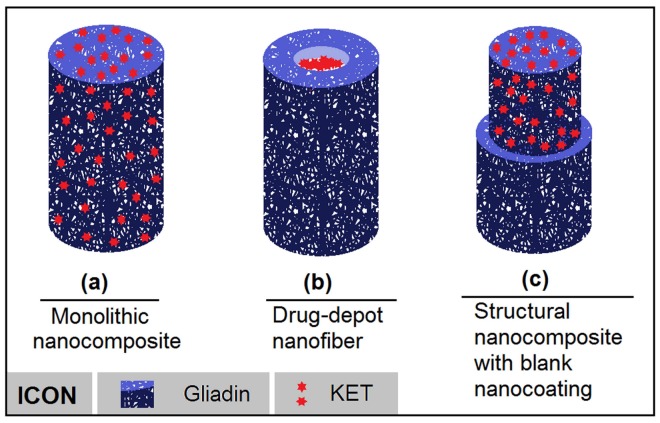
Drug distributions in electrospun nanofibers: (**a**) homogeneous; (**b**) drug depots in the core section; and, (**c**) blank nanocoating on core nanocomposites.

In medicated nanomaterials, drug distribution constantly influences the drug release profile, particularly for poor water-soluble drugs, whose amorphous state is important for achieving drug controlled release profiles [43,44]. In this study, the nanocoating significantly altered the drug distribution. Figure 8 shows the two kinds of drug distribution characteristics in the core part of core-heath nanofibers. One type is drug depots (Figure 8b), in which the pure drug was concentrated in the core parts of nanofibers, as demonstrated by our previous work [26,33]. The other type involves the drug-carrier nanocomposite cores that are disclosed in this study. The advantages of this new structure can be concluded as follows: (1) drug distribution is absent in nanofiber surfaces compared with traditional monolithic nanofibers (Figure 8a); (2) the blank nanocoating can be exploited as a key parameter to manipulate the sustained release of inner drug molecules; (3) the drug is present in an amorphous state and easily dissolves in water when compared with drug depots (Figure 8b); (4) a drug-polymer composite should be more stable than pure drugs, and its corresponding working fluid is easier to process than pure drug solutions using electrospinning [45]. Correspondingly, this new structure has generated unusual functional performances, changing the drug release kinetics from the traditional exponential model to the desired zero-order model, eliminating initial burst release, prolonging sustained release period, and releasing 95.7 ± 4.7% of loaded cargoes in a linear manner. 

## 5. Conclusions

In this study, a modified coaxial process was successfully carried out, where dilute gliadin solution without electrospinnability was explored as the sheath working fluid to encapsulate the core KET-gliadin electrospinnable solution. A novel core-sheath nanostructure was created through the new process, in which a thin blank nanocoating, with a thickness of about 25 nm, formed as the sheath section on the core KET-loaded gliadin nanocomposite. Similarly with monolithic nanocomposites (F1), KET was distributed in the gliadin matrices in an amorphous state in the core sections of nanofibers F2, reflecting that the new process exerted minimal influence on the physical state of the active ingredient. In vitro dissolution tests demonstrated that the thin nanocoating significantly changed the drug sustained release behavior from a typical kinetic exponent equation for nanofibers F1 to a zero-order linear equation for nanofibers F2. The novel structure of nanocoating can ensure a better sustained release over a period of 16 h to release 95.7% of the loaded cargoes in a linear manner. The present protocol paves a new way for developing nanomaterials with special nanostructural characteristics to achieve the designed functional performances.
